# Effects of Focal Vibration over Upper Limb Muscles on the Activation of Sensorimotor Cortex Network: An EEG Study

**DOI:** 10.1155/2019/9167028

**Published:** 2019-05-27

**Authors:** Wei Li, Chong Li, Quan Xu, Linhong Ji

**Affiliations:** ^1^Division of Intelligent and Biomechanical System, State Key Laboratory of Tribology, Department of Mechanical Engineering, Tsinghua University, Haidian, Beijing, China; ^2^Department of Rehabilitation Medicine, Beijing Tsinghua Changgung Hospital, School of Clinical Medicine, Tsinghua University, Beijing, China

## Abstract

Studying the therapeutic effects of focal vibration (FV) in neurorehabilitation is the focus of current research. However, it is still not fully understood how FV on upper limb muscles affects the sensorimotor cortex in healthy subjects. To explore this problem, this experiment was designed and conducted, in which FV was applied to the muscle belly of biceps brachii in the left arm. During the experiment, electroencephalography (EEG) was recorded in the following three phases: before FV, during FV, and two minutes after FV. During FV, a significant lower relative power at C3 and C4 electrodes and a significant higher connection strength between five channel pairs (Cz-FC1, Cz-C3, Cz-CP6, C4-FC6, and FC6-CP2) in the alpha band were observed compared to those before FV. After FV, the relative power at C4 in the beta band showed a significant increase compared to its value before FV. The changes of the relative power at C4 in the alpha band had a negative correlation with the relative power of the beta band during FV and with that after FV. The results showed that FV on upper limb muscles could activate the bilateral primary somatosensory cortex and strengthen functional connectivity of the ipsilateral central area (FC1, C3, and Cz) and contralateral central area (CP2, Cz, C4, FC6, and CP6). These results contribute to understanding the effect of FV over upper limb muscles on the brain cortical network.

## 1. Introduction

In the past few years, more effort has been paid to studying the effects of focal vibration (FV) at a high frequency (50∼120 Hz) and with a low amplitude on the rehabilitation of neurological diseases, such as stroke, spinal cord injury, multiple sclerosis, and cerebral palsy [1]. As for patients with stroke, FV can improve various abilities and functionalities, including walking [2], postural sway and gait ability [3], motor performances of reaching movement [4], stability of the proximal arm [5], and reducing spasticity [6, 7]. As for patients with spinal cord injury, FV reduced spasticity [1] and elicited stepping movements [8]. Additionally, research indicated FV can also contribute to the improvement of movement control in patients with multiple sclerosis [9].

The neurophysiological mechanism underlying how FV benefits the recovery of motor function for patients with neurological diseases has been explored mainly in experiments with healthy subjects. At spinal cord level, some studies have shown that FV induced the firing of muscle spindle primary endings (Ia afferent fibers) [10–12]. At the cortical level, studies using transcranial magnetic stimulation (TMS) showed that FV enhanced the excitability of motorcortical representation of vibrated muscle [6, 13]. Besides, some researchers also have proved that vibrotactile stimulation on the palm or fingers caused the activation of primary motor cortex (M1), primary somatosensory cortex (S1), and secondary somatosensory cortex (S2) using functional magnetic resonance imaging (fMRI) [14–18]. However, how FV applied to other body sites influence the activation of sensorimotor cortex has not been fully studied.

Electroencephalography (EEG) is an electrophysiological method to record the electrical activity of the brain. The rolandic alpha rhythm (sensorimotor “mu” rhythms) concentrates mainly in the somatosensory postcentral gyrus, while the rolandic beta rhythm (sensorimotor beta rhythms) mainly originates in the precentral motor cortex [19, 20]. It was well accepted that event-related desynchronization (ERD) (the decrease of power) over the sensorimotor areas represented the activation of the sensorimotor cortex. On the contrary, event-related synchronization (ERS) (the increase of power) reflected the deactivation of the sensorimotor cortex [21, 22]. Some EEG-fMRI studies also showed that the decrease of EEG power was related to activation of the sensorimotor cortex [19, 23–25]. As for functional connectivity, it can reflect the level of synchronization between the signals of different scalp regions, as well as the topological and dynamics properties of information flow between different brain areas [26, 27]. Recently, it has been used as a biomarker to investigate the mechanism of functional recovery in patients with neurological diseases [28–31].

In this study, we aimed to study the effect of FV applied over left biceps brachii on the sensorimotor cortex during FV and after FV. We recorded the EEG activity before FV, during FV, and 2 min after FV. Then, we analyzed the changes of relative power at C3 and C4 and functional connectivity of the central region (FC5, FC1, FC2, FC6, C3, Cz, C4, CP5, CP1, CP2, and CP6) in the alpha (8–12 Hz) and beta (12.5–30 Hz) band.

## 2. Materials and Methods

### 2.1. Participants

Twenty male right-handed healthy subjects with a mean age of 26 years (±0.6 years) were recruited in Tsinghua University in this study. All subjects were informed about the procedure of the experiment. All the subjects gave written consent prior to the experiment. The study was approved by the institutional ethics committee.

### 2.2. Experimental Setup

**Figure 1:**
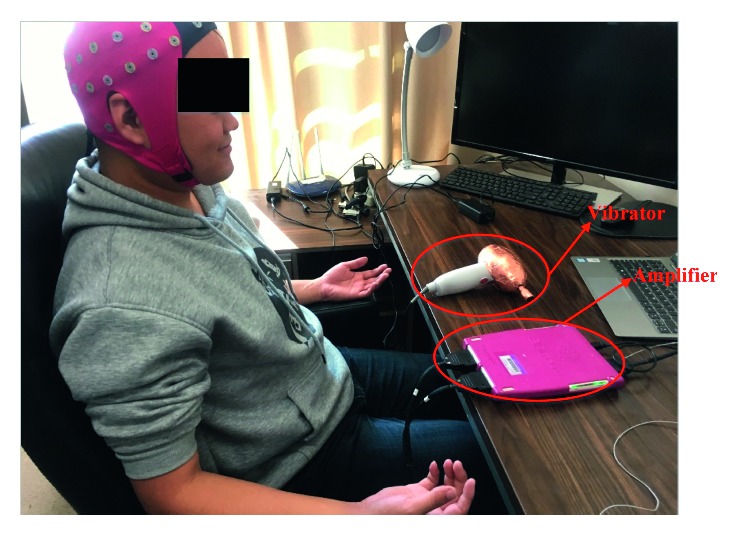
Illustration of the experimental setup.

All the participants were informed to have a good rest one day before the experiment in order to minimize drowsiness. During the experiment, each subject was seated in a comfortable chair with both arms on the armrest and their hand supinated so that their upper limbs were relaxed. FV with frequency at 75 Hz and amplitude at 1.2 mm was produced by a mechanical vibration device with a vibration head (YS-889, Jialemei Health Care Co., Ltd., Taiwan, China), as shown in Figure 1. The mechanical vibration device was operated at a power frequency of 50 Hz. FV was applied perpendicularly over the muscle belly of biceps brachii in the left arm lasting for three minutes. During the EEG recording, subjects were asked to minimize head movement, eye movement, body movement, and chewing and were required to wear earplugs to reduce external noise and vibration noise. Based on the recommendation to use eyes-closed resting EEG as a baseline for tasks without visual stimuli [32], all the subjects were asked to keep their eyes closed during this experiment. Specifically, EEG was collected in the following three phases: (1) before FV (baseline: before-FV), the resting state EEG was recorded with the eyes closed for 4 min; (2) during FV (during-FV), EEG were recorded with the eyes closed for 3 min of vibration; and (3) after FV (after-FV), EEG were recorded with the eyes closed for 3 min, starting at 2 minutes after the termination of FV.

### 2.3. EEG Recordings

EEG signals were recorded with ANT hardware and software (B.V., Enschede, the Netherlands) from 32 Ag/AgCl electrodes mounted in a commercial WaveGuard EEG Cap (Eemagine Medical Imaging Solutions GmbH, ANT Advanced Neuro Technology) and positioned over the whole scalp according to the international 10–20 system, as well as two electrodes on the left and right mastoids. A ground reference electrode was located between the Fpz and Fz electrode, and the reference electrode was located at the Cpz electrode. The sampling rate was set at 1000 Hz. Electrode impedances were kept below 5 kΩ.

### 2.4. EEG Signal Analysis

#### 2.4.1. Signal Preprocessing

The EEG signals were preprocessed in EEGLAB 14 (EEGLAB toolbox, Swartz Center for Computational Neurosciences, La Jolla, CA; http://www.sccn.ucsd.edu/eeglab). Data were divided into segments of 2 s. Artifacts were visually detected. The data were referenced to the common average reference. The power line noise 50 Hz was removed. A band-pass filter was set between 0.5 Hz and 50 Hz. The Welch method (pWelch algorithm, an overlapping 1-second hanning window, no phase shift) was applied to compute the power spectral density of each epoch (2 second duration, 2000 data points) using a 0.5 Hz frequency resolution, and all the epochs were then averaged.

#### 2.4.2. Relative Power Analysis

In this study, relative power was estimated in the two frequency bands: alpha (8–12 Hz) and beta (12.5–30 Hz). The relative power RP(·) was calculated as follows:

(1) $$\begin{array}{c} \text{RP}\left(f_{1},f_{2}\right)=\frac{\int_{f_{1}}^{f_{2}}\text{PSD}\left(f_{1},f_{2}\right)df}{\int_{f_{1}}^{f_{2}}\text{PSD}\left(0.5,50\right)df}, \end{array}$$

where *f*_1_ and *f*_2_ indicate the low and high frequency of the specified frequency band, respectively. PSD(·) indicates the power spectral density.

Alpha and beta motor-related power desynchronizations (MRPD) at C3 and C4 electrodes were used to indicate the activation of primary sensorimotor cortex [33]. MRPP was calculated as follows:

(2) $$\begin{array}{c} {\text{MRPD}}_{\text{during}‐\text{FV}}=\frac{{\text{RP}}_{\text{during}‐\text{FV}}-{\text{RP}}_{\text{baseline}}}{{\text{RP}}_{\text{baseline}}}, \end{array}$$

(3) $$\begin{array}{c} {\text{MRPD}}_{\text{after}‐\text{FV}}=\frac{{\text{RP}}_{\text{after}‐\text{FV}}-{\text{RP}}_{\text{baseline}}}{{\text{RP}}_{\text{baseline}}}, \end{array}$$

where RP_baseline_, RP_during‐FV_, and RP_after‐FV_ indicate the relative power before FV, during FV, and after FV, respectively. The negative or positive values reflected alpha (beta) movement-related power desynchronization or synchronization, respectively.

#### 2.4.3. Functional Connectivity Analysis

In this study, functional connectivity was estimated using imaginary coherence, which reduced overestimation biases that exist in many other measures, such as phase locking value, absolute coherence, and synchronization likelihood [34–36]. Due to the common reference, cross-talk, and volume conduction, these measures generated spurious interactions with no time lag. Imaginary coherence was expressed as the imaginary part of coherency *C*_*ij*_(*f*), which was defined as the normalized cross-spectrum:

(4) $$\begin{array}{c} C_{ij}\left(f\right)=\frac{S_{ij}\left(f\right)}{{\left(S_{ii}\left(f\right)S_{jj}\left(f\right)\right)}^{1/2}}, \end{array}$$

where *S*_*ij*_(*f*) ≡ 〈*x*_*i*_(*f*)*x*_*j*_^*∗*^(*f*)〉 is the cross-spectrum. *x*_*i*_(*f*) and *x*_*j*_(*f*) indicate the complex Fourier transforms of the time series ${\hat{x}}_{i}\left(t\right)$ and ${\hat{y}}_{j}\left(t\right)$ of the channel *i* and *j*, respectively. 〈〉 indicates expectation value and *∗* indicates complex conjugation. Expectation value was estimated as an average over all the segments.

In this study, the functional connectivity of the central region (FC5, FC1, FC2, FC6, C3, Cz, C4, CP5, CP1, CP2, and CP6) was calculated.

### 2.5. Statistical Analysis

The statistical analysis was performed by SPSS Statistics 20. In the analysis of relative power, two-way repeated measures analysis of variance (ANOVA) was used. Before two-way repeated ANOVA was performed, the Shapiro–Wilk test was used to determine if all the data sets of relative power were well-modeled by a normal distribution. The ANOVA factors included the main factor condition (before FV, during FV, and after FV) and the main factor electrode (C3 and C4). If the main factor condition or electrode showed a significance, a paired-sample *t*-test was then performed. In the statistical analysis of functional connectivity, there were a large number of tested channel pairs (*C*_11_^2^=55). To make sure the probability of one or more null hypotheses incorrectly rejected, false discovery rate (FDR) correction was performed. The Type I error was set to 0.05. Pearson's correlation analysis was performed between alpha MRPD and beta MRPD during and after FV.

## 3. Results

### 3.1. Relative Power Analysis

**Figure 2:**
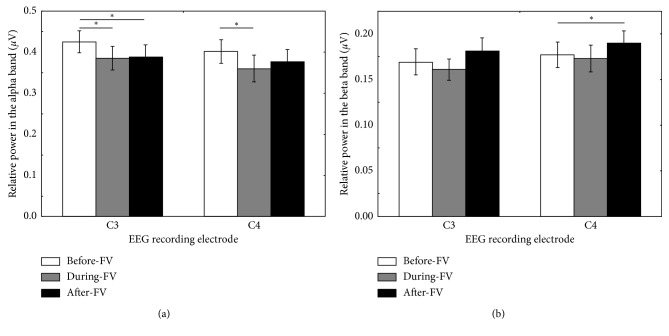
Relative power of the alpha band (a) and beta band (b) at C3 and C4 before FV (white), during FV (grey), and after FV (black). Note: mean values of relative power with SEM are present in charts; asterisks indicate significant differences (*p* < 0.05).

In the alpha band, the Shapiro–Wilk test showed relative power at C3 (*p*=0.991, 0.867,  *and* 0.438) and C4 (*p*=0.126, 0.097,  *and* 0.156) during three phases, before FV, during-FV, and after-FV was normally distributed. Two-way repeated-measures ANOVA showed the interaction electrode × condition (*F*(2,38)=1.137; *p*=0.331) and the main factor electrode (*F*(1,19)=2.480; *p*=0.132) were not significant, while the main factor condition (*F*(2,38)=4.718; *p*=0.015) was significant. A paired-sample *t*-test for the relative power at C3 and C4 showed a significant decrease during FV (one tailed: *p*=0.0095 *and* 0.0075) and after FV (one tailed: *p*=0.0145 *and* 0.037) compared to before-FV. After the Bonferroni correction, the relative power of C3 and C4 during FV showed a significant decrease (one tailed: *p*=0.019 *and* 0.015), whereas the relative power of C3 after FV showed a significant decrease (one tailed: *p*=0.029) (Figure 2).

In the beta band, the Shapiro–Wilk test showed relative power at C3 (*p*=0.192, 0.709,  *and* 0.510) and C4 (*p*=0.348, 0.055,  *and* 0.923) during three phases, before-FV, during-FV, and after-FV, was normally distributed. Two-way repeated-measures ANOVA revealed that the interaction electrode × condition (*F*(2,38)=0.323; *p*=0.726) and the main factor electrode (*F*(1,19)=2.135; *p*=0.160) were not significant, while the main factor condition (*F*(2,38)=4.818; *p*=0.014) was significant. The paired-sample *t*-test for the relative power at C3 and C4 showed a significant increase after FV compared to before-FV (one tailed: *p*=0.033 *and* 0.017). After the Bonferroni correction, the relative power of C4 after FV showed a significant increase compared to before-FV (*p*=0.034), as shown in Figure 2.

### 3.2. Pearson's Correlation Analysis

**Figure 3:**
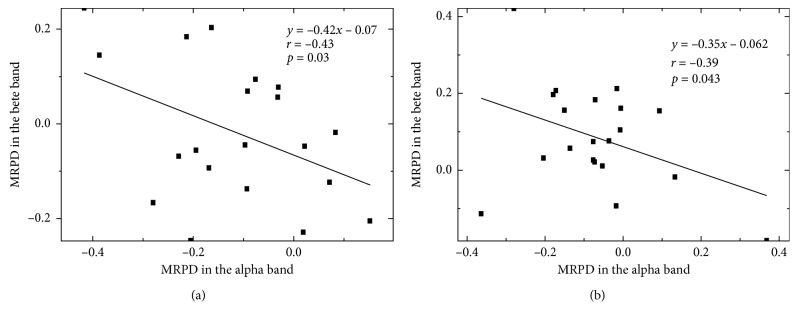
Pearson's correlation between alpha MRPD and beta MRPD during FV (a) and after FV (b) at C4. Solid squares indicate each subject's MRPD.

The values of MRPD at C3 and C4 in the alpha and beta bands are shown in Figure 3. A significant negative correlation was found between MRPD at C4 in the alpha band and one in the beta band during FV (*p*=0.03, Pearson's *r* = −0.43), as well as after FV (*p*=0.043, Pearson's *r* = −0.39) (Figure 3). No significant correlation was found between MRPD at C3.

### 3.3. Functional Connectivity Analysis

**Figure 4:**
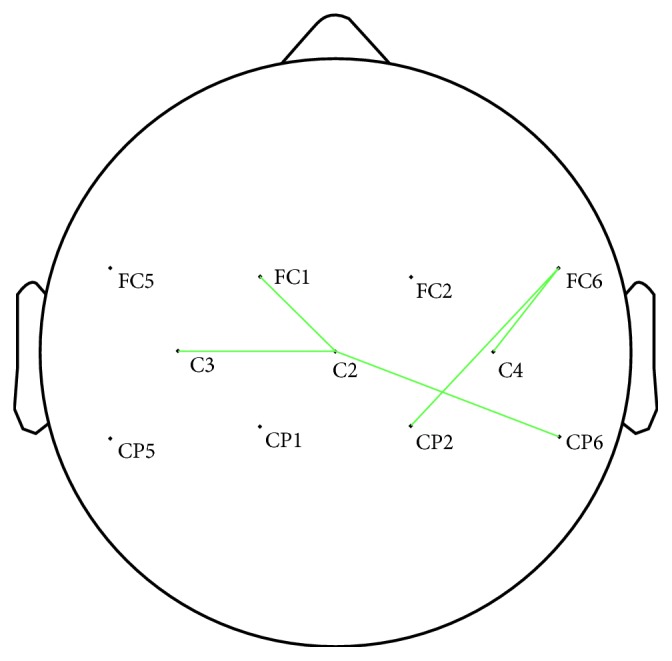
Connection strength of different channel pairs with significant differences during FV in the alpha band after FDR correction.

After FDR correction tests, the connection strength of Cz-CP6, Cz-FC1, Cz-C3, C4-FC6, and FC6-CP2 in the central region showed a significant increase in the alpha band during FV, which is shown in Figure 4. The *p* values of connection strength of five channel pairs were 0.0093, 0.0259, 0.0261, 0.0047, and 0.0351, respectively.

## 4. Discussion

In order to investigate the effect of FV applied over upper limb muscles on sensorimotor cortex during FV and after FV, the experiment was conducted in which FV was delivered to the muscle belly of biceps brachii in the left arm and EEG was monitored in three phases, before FV, during FV, and after FV. The results showed that FV on upper limb muscles could activate bilateral S1 and strengthen connection strength of the central region, including Cz-FC1, Cz-C3, Cz-CP6, C4-FC6, and FC6-CP2. The effect could not be maintained two minutes after FV. We also find that the changes of relative power at C4 in the alpha band have a negative correlation with the ones in the beta band during FV and after FV.

### 4.1. Before FV and during FV

In the present study, the results show that the application of FV over the muscle belly of biceps brachii in the left arm can activate contralateral S1. The “mu” rhythms originate mainly in the somatosensory postcentral gyrus [19, 20]. Moreover, the previous studies found that desynchronized power in the alpha band had a positive correlation with the activation of S1 [20, 23–25]. The present result was in line with the previous studies showing that vibrotactile at the palm or finger could activate contralateral S1 using fMRI [14, 16, 18]. It also meant that FV, which was similar to motor preparation, motor execution, motor imagery, and somatosensory stimuli, induced alpha-ERD pattern [22, 37, 38]. There was a consensus that muscle spindle and cutaneous mechanoreceptors, like Merkel afferents, Meissner afferents, and Pacinian afferents, responded to FV at the frequency of 75 Hz applied over muscle belly [39]. Based on this, it could be inferred that the activation of S1 could result from two pathways. One way is that the proprioceptive information from muscle spindle travels along the upper body proprioceptive pathway, which decussates in the caudal medulla through the posterior column-medial lemniscus pathway, finally to reach S1 through the ventral posterior lateral nucleus of the thalamus. The other pathway is that this cutaneous mechanoreceptor information conveys by a separate set of first-order neurons located in the trigeminal ganglion, then ascends to the ventral posterior medial nucleus of the thalamus through the neurons given off by the trigeminal brainstem nuclei, and finally reaches S1 [39]. Besides, the present result shows the occurrence of the activation of ipsilateral S1 following FV, which could originate from the input of about 10% uncrossed corticospinal tracts [40]. The activation of ipsilateral S1 following FV is also found in several studies [14, 15].

In the present study, the connection strength of Cz-FC1, Cz-C3, Cz-CP6, C4-FC6, and FC6-CP2 can be strengthened following FV. Some studies showed that C3 and C4 could project close to postcentral gyrus whilst Cz and FC1 could project close to precentral gyrus [35, 41, 42]. It seemed that the connection strength between ipsilateral postcentral gyrus and precentral gyrus is strengthened, which is similar to earlier findings indicating that functional connectivity of the ipsilateral sensorimotor area during real movements was strengthened [43].

### 4.2. Before FV and after FV

After FV, the present study shows that the relative power at C4 in the beta band has a statistically significant increase, which indicates the rebound of beta power after FV. It has been generally accepted that beta rebound coincided with reduced excitability of motor cortex neurons, which is similar to the phenomenon after active movement, passive movement, motor imagery, and somatosensory stimulation [21, 44–46]. Based on “functional inhibition” hypothesis, a desynchronized alpha band following the occurrence of a synchronized power in the beta band could reflect a mechanism of functional inhibition of the motor cortex by somatosensory processing [47]. It could be inferred that the activation of somatosensory cortex induced by FV could inhibit the excitability of motor cortex after FV. In the present study, the occurrence of alpha MRPD and beta MRPD at C4 shows a significant negative correlation between during-FV and after-FV. It is in line with the previous result showing that a stronger mu rhythm ERD appeared with an enhanced beta ERS with foot movement [22]. Besides, no significant reduction of relative power at C4 in the alpha band showed that the excitability of S1 was not maintained about two minutes after FV.

The differences between our findings and the current literature are that M1 is not activated and the duration of the excitability of sensorimotor areas induced by FV is shorter in this study. In the previous fMRI studies, FV applied at the hand palm or finger could activate M1 [14, 15, 18]. On the one hand, the number of muscle spindles per gram of muscle tissue at other sites of limb muscles was lower than at the hand palm (130/g) [14, 48]. The proprioceptive input is too weak to induce the activation of M1. One the other hand, it might be ascribed to the difference in the amplitude of FV. One study showed that vibration stimulus with low amplitude (0.4 mm) activated sensory and motor cortex more strongly than high amplitude (1.6 mm) [17]. In addition, the duration of effect could be related to the lasting time of FV. There were several studies using TMS showing that the excitability of sensorimotor cortex was not enhanced shortly after short-lasting FV [49–51], whilst the excitability of sensorimotor cortex lasted for longer time after long-lasting vibration stimulation [52]. Recently, one study also showed that FV could increase the excitability of S1-M1 immediately after FV, but the time of FV lasted for about thirty minutes [33].

Notably, our results show that FV could activate S1 and strengthen the functional connectivity of the sensorimotor system. The activation of S1 could play an important role in the recovery of motor function in patients with neurological diseases. On the one hand, somatosensory input from FV to the motor cortex, via corticocortical connections with S1, plays a critical role in motor relearning after hemiparetic stroke [53, 54]. On the other hand, FV, as one of the most effective modulators of cortical structure and function, could modify synaptic efficacy and transmission and as a consequence causes a cortical reorganization of the somatosensory representational maps. The reorganization of function and structure could contribute to the recovery of limb motor function. In the present study, the results using EEG are similar to ones using fMRI. It could be an appropriate way for EEG to further investigate the underlying neurophysiological mechanisms of FV on rehabilitation of motor function.

One important limitation of this study is that this is a pilot study, but not a randomized controlled trial. It cannot be precluded that part of the effects occurred could be due to the experimental procedure and not due to vibration stimulation. Nevertheless, our findings indicate that FV could activate the sensorimotor cortex and strengthen the connection strength between central regions by a comparison of before-FV with during-FV. It can be feasible to explore the effect of FV on sensorimotor cortex using EEG for later use on clinical research. In a future clinical experiment, a randomized controlled trial will be designed to explore the mechanism of FV in motor rehabilitation using EEG.

## 5. Conclusion

Our study shows that FV on upper limb muscles could activate the activity of primary somatosensory cortex and strengthen connection strength of the central region. Based on our present results, we will investigate the effect of FV on the activation of sensorimotor cortex for stroke patients, to further explore the underlying neurophysiological mechanisms of FV on rehabilitation of motor function in the clinical experiment.

## Data Availability

The EEG data used to support the findings of this study are available from the corresponding author upon request.
